# Development and validation of a scoring system for the prediction of HIV drug resistance in Hubei province, China

**DOI:** 10.3389/fcimb.2023.1147477

**Published:** 2023-05-10

**Authors:** Jisong Yan, Wenyuan Zhang, Hong Luo, Xianguang Wang, Lianguo Ruan

**Affiliations:** ^1^ Department of Respiratory and Critical Care Medicine, Wuhan Jinyintan Hospital, Tongji Medical College of Huazhong University of Science and Technology, Hubei Clinical Research Center for Infectious Diseases, Wuhan Research Center for Communicable Disease Diagnosis and Treatment, Chinese Academy of Medical Sciences, Joint Laboratory of Infectious Diseases and Health, Wuhan Institute of Virology and Wuhan Jinyintan Hospital, Chinese Academy of Sciences, Wuhan, Hubei, China; ^2^ Department of Infectious Diseases, Wuhan Jinyintan Hospital, Tongji Medical College of Huazhong University of Science and Technology, Hubei Clinical Research Center for Infectious Diseases, Wuhan Research Center for Communicable Disease Diagnosis and Treatment, Chinese Academy of Medical Sciences, Joint Laboratory of Infectious Diseases and Health, Wuhan Institute of Virology and Wuhan Jinyintan Hospital, Chinese Academy of Sciences, Wuhan, Hubei, China

**Keywords:** HIV/AIDS, antiretroviral therapy, drug resistance, scoring system, predicting model

## Abstract

**Objective:**

The present study aimed to build and validate a new nomogram-based scoring system for the prediction of HIV drug resistance (HIVDR).

**Design and methods:**

Totally 618 patients with HIV/AIDS were included. The predictive model was created using a retrospective set (N = 427) and internally validated with the remaining cases (N = 191). Multivariable logistic regression analysis was carried out to fit a model using candidate variables selected by Least absolute shrinkage and selection operator (LASSO) regression. The predictive model was first presented as a nomogram, then transformed into a simple and convenient scoring system and tested in the internal validation set.

**Results:**

The developed scoring system consisted of age (2 points), duration of ART (5 points), treatment adherence (4 points), CD4 T cells (1 point) and HIV viral load (1 point). With a cutoff value of 7.5 points, the AUC, sensitivity, specificity, PLR and NLR values were 0.812, 82.13%, 64.55%, 2.32 and 0.28, respectively, in the training set. The novel scoring system exhibited a favorable diagnostic performance in both the training and validation sets.

**Conclusion:**

The novel scoring system can be used for individualized prediction of HIVDR patients. It has satisfactory accuracy and good calibration, which is beneficial for clinical practice.

## 1 Introduction

Antiretroviral therapy (ART) has decreased global mortality and morbidity, while also improving the life expectancy of people living with HIV (PLWH) (GBD 2017 HIV collaborators, 2019; Koay et al., 2021). Initially, ART was required to be used only in immunologically suppressed patients with CD4 counts <200 cells/mm^3^, but has been applied to all PLWH regardless of CD4 count since 2016 (World Health Organization, 2015; World Health Organization, 2016; Koay et al., 2021). However, with the popularity of ART, the issue of HIV drug resistance is receiving more and more attention. HIV, as an RNA virus, is characterized by an increased error rate during reverse transcription of the RNA helix, producing strains of the virus with drug-resistant mutations. This is defined as acquired HIV drug resistance (ADR) (Wei et al., 1995; McCluskey et al., 2019; Giacomelli et al., 2020). In addition, ADR variants can transmit the mutated virus to untreated individuals, resulting in transmitted HIV drug resistance (TDR) (Zhukova et al., 2017; Blassel et al., 2021; World Health Organization, 2021b).

HIV drug resistance (HIVDR) is one of the problems in combating the HIV epidemic, elevating the risk of PLHIV whose disease continues to be infectious. In 2021, the Joint United Nations Programme on HIV/AIDS (UNAIDS) released the “Fast-Track strategy” initiative with the following goals. By the year 2030, there is a global consensus to aim for 95% of all PLWH knowing this diagnosis, 95% of those diagnosed receiving treatment, and 95% of those on treatment achieving sustained virologic suppression (Joint United Nations Programme on HIV/AIDS, 2015; Collier et al., 2019). For these goals, we need not only HIV drugs with durable efficacy, tolerability and safety (Mbhele et al., 2021), but also a rapid and convenient screening testing strategy for people at high risk with DRM.

At present, there are many methods for drug resistance gene detection, including Illumina NGS, Sanger population sequencing, AS-PCR, 454 pyrosequencing, and other new approaches (Mbunkah et al., 2020; Metzner, 2022; Pyne et al., 2022). However, assays assessing HIV genotypic and phenotypic drug resistance are more complex, with problems such as high testing cost and no uniform quality index guidance (Harrigan and Côté, 2000; Hirsch et al., 2008). Meanwhile, despite the relatively elevated diagnostic accuracy, the above methods could not be applied in most hospitals, especially primary hospitals, due to high cost and strict requirements for medical equipment.

In recently years, the creation of mathematical models based on various markers has been increasingly used in medicine with the development of analytical methodologies. Unfortunately. previous studies have focused only on risk factors for drug resistance, using a single indicator with poor predictive power. Several studies have demonstrated that HIV-1 is prone to drug resistance mutations (DRMs) due to prolonged ART exposure and poor adherence, leading to viral rebound and treatment failure (Larder and Kemp, 1989; Rhee et al., 2020; Blassel et al., 2021; Koay et al., 2021). In addition, the extent of DRM may also depend on individual characteristics, the type of regimen, baseline CD4^+^ T cell count and viral load (Karade et al., 2018). As proven by these studies, the occurrence of HIVDR varies from patient to patient, which illustrates the importance of developing and using risk prediction models for HIV drug resistance.

Therefore, our main objective was to generate a multivariable logistic regression prediction model based on a mixture of clinical factors to predict HIVDR. Then, a unique scoring system was build using the primary prediction model’s modified nomogram for easy clinical application. Additionally, in the retrospective analysis, we internally verified the diagnostic value of the improved scoring model.

## 2 Materials and methods

### 2.1 Study design and participants

The study was a retrospective HIV/AIDS cohort collected from the AIDS Prevention and Control Information System (AIDS-PCIS). We incorporated patients with HIV/AIDS registered in four regions of Hubei province, including Wuhan, Huangshi, Jingmen and Xianning, from June 2017 to June 2022. Totally, 618 PLWH were enrolled and divided into two groups. 70% of participants (N=427) were randomly allocated to the training set and the remaining 30% (N=191) in the validation set. We performed genotypic drug resistance testing in all participants. The sequences were sent to the Stanford HIV Drug Resistance Database (http://hivdb.stanford.edu) to be evaluated for antiretroviral resistance using the list of major HIV-1 resistance mutations (major HIV-1 drug resistance mutations) standardized by the Stanford HIV Database (http://hivdb.stanford.edu/assets/media/resistance-mutation-handout feb2019.b0204a57.pdf). We defined patients who were potentially resistant or differentially resistant to any of the antiretroviral drugs as the drug-resistant (DR) group, and patients who were drug-sensitive as the drug-sensitive (DS) group.

Inclusion criteria were: (1) confirmed as a PLWH; and (2) ≥18 years old. Exclusion criteria were: (1) pregnancy and lactation in women; (2) refusal of drug resistance testing. Variables of PLWH including age, sex, body mass index (BMI), transmission route, duration of ART, adherence, CD4 T-cell count, HIV viral load and laboratory parameters were collected before the drug resistance testing. Treatment adherence was assessed by the proportion of days covered, which is the sum of days during follow-up. Poor adherence was defined as the proportion of days covered less than 80%. HIV viral load was logarithmically transformed before being included. The Wuhan Jinyintan Hospital’s ethics committee approved the study protocol, and all participant data were obtained anonymously.

### 2.2 Statistical analysis

Although we did not officially compute sample size, we evaluated the data sufficiency using the event per variable method (Moons et al., 2015). We used multiple imputation by chained equation (mice) package to impute missing values of baseline parameters (Zhang, 2016). Continuous and categorical data were presented as number (percentage, %) and median (interquartile range, IQR), respectively. Group comparisons between drug resistance (DR) and drug sensitive (DS) participants were performed by the Mann-Whitney U test, Chi square test or Fisher’s exact test, as suitable. Additionally, the best effective predictors were selected by the Least Absolute Shrinkage and Selection Operator (LASSO) technique. Then, the variables extracted were assessed by multivariable logistic regression analysis (MLRA) to construct a predictive model. With the independent variables obtained in the MLRA, a scoring system was created based on a nomogram using the RMS package in R. The scores of each variable and individual were obtained by the following steps: ①Variables scoring: the highest scoring variable in the MLAR was designated as 10 points (HIV viral load *7 in this study). Scores for the other variables were calculated by equating them according to the model coefficients and rounded to integers at the last. ②Patients scoring: the scores of each patient were the sum of the scores of all variables in the scoring system. The predictive power of the scoring system was evaluated by computing the area under the curve (AUC). The Hosmer-Lemeshow test was used to assess the model’s calibration, and decision curve analysis was employed to evaluate its clinical utility (DCA). Another independent dataset was employed for further validation. All analyses were conducted with SPSS version 26.0 (IBM Inc., Chicago, IL, USA) and R Project version 4.2.0 (http://cran.r-project.org). Two-sided p<0.05 was considered statistically significant.

## 3 Results

### 3.1 Study population

**Figure 1:**
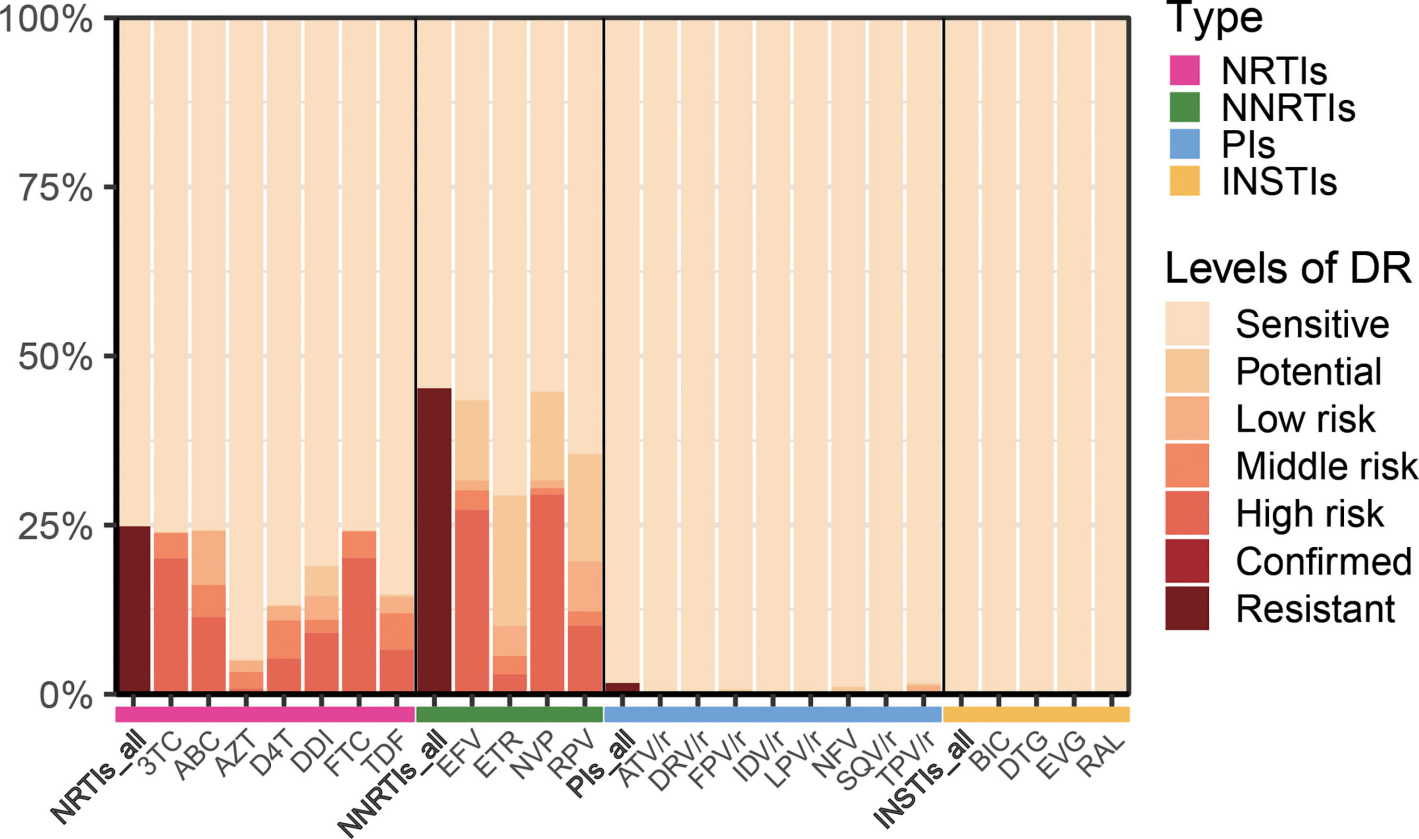
Levels of resistance to different types of antiretroviral drugs. NRTIs, nucleoside reverse-transcriptase inhibitors; NNRTIs, non-nucleoside reverse-transcriptase inhibitors; PIs, protease inhibitors; INSTIs, integrase inhibitors; 3TC, lamividine; ABC, abacavir; AZT, zidovudine; D4T, stavudine; DDI, didanosine; FTC, emtricitabine; TDF, tenofovir disoproxil; EFV, efavirenz; ETR, etravirine; NVP, nevirapine; RPV, rilpivirine; ATV/r, atazanavir/ritonavir; DRV/r, darunavir/ritonavir; FPV/r, fosamprenavir/ritonavir; IDV/r, indinavir/ritonavir; LPV/r, lopinavir/ritonavir; NFV, nelfinavir; SQV/r, saquinavir; TPV/r, tipranavir; BIC, bictegravir; DTG, dolutegravir; EVG, elvitegravir; RAL, raltegravir.

In this study, of the 618 patients included, most were male (83.2%, 514/618), and mean patient age was 39.5 years (IQR: 24.0-55.0 years). HIV drug resistance testing were performed in 303 patients before starting ART therapy, while the remaining 315 patients were tested after more than 6 months of ART therapy. The major infection route was sexual transmission: heterosexual transmission (50.2%) and MSM (48.1%). Among the DRMs detected, antiretroviral resistance was observed in 47.4% (293/618) of PLWH undergoing ART therapy (**Table 1**). As presented in **Figure 1**, NNRTIs showed elevated frequencies of at least one primary mutation, i.e., in 45.1% (279/618) of patients, while for NRTIs and PIs, these frequencies were 24.5% (152/618) and 1.6% (10/618), respectively.

**Table 1:** Baseline characteristics.

Variables	Training set(N=427)			Validation set(N=191)			P-value
	DR group N=207	DS group N=220	P-value	DR group N=86	DS group N=105	P-value	
Age, year							
<28y	86(41.5%)	59(26.8%)		24(27.9%)	27(25.7%)		
≥28y	121(58.5%)	161(73.2%)	0.010	62(72.1%)	78(74.3%)	0.072	0.314
Gender Female male	40(19.3%) 167(80.7%)	30(13.6%) 190(86.4%)	0.113	15(17.4%) 71(82.6%)	19(18.1%) 86(81.9%)	0.907	0.666
BMI, kg/m2	21.0(19.2-23.8)	22.4(20.1-24.6)	0.012	21.5(19.6-23.18)	21.72(19.64-23.58)	0.587	0.028
Infection route							
Heterosexual	102(49.3%)	100(45.5%)	0.483	51(59.3%)	57(54.3%)	0.591	0.065
MSM Others	100(48.3%) 5(2.4%)	117(53.2%) 3(1.4%)		33(38.4%) 2(2.4%)	47(44.8%) 1(0.9%)		
Regular sex partner(s)							
No Yes	130(62.8%) 77(37.2%)	153(69.5%) 67(30.5%)	0.141	52(60.5%) 34(39.5%)	75(71.4%) 30(28.6%)	0.110	<0.001

MSM: men who have sex with men; Other Infection route: drug injection, blood transmission, unknow; BMI: body mass index; Regular sex partner(s)：including married or with a regular sexual relationship.

### 3.2 Construction of nomogram and scoring system

**Figure 2:**
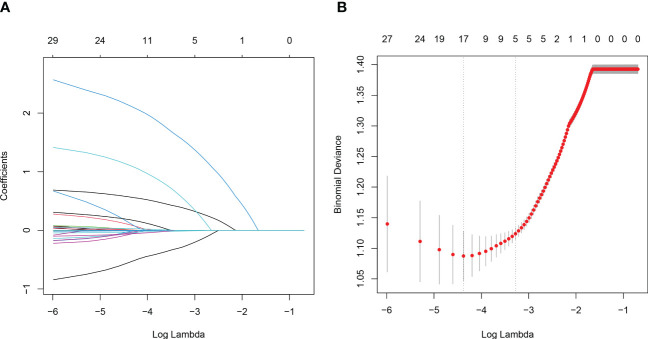
Variables selection using the LASSO regression and multivariable regression analysis. **(A)** Tuning parameter (lambda) selection of deviance in the LASSO regression based on the minimum criteria (left dotted line) and the 1-SE criteria (right dotted line). **(B)** A coefficient profile plot was created against the log (lambda) sequence.

**Figure 3:**
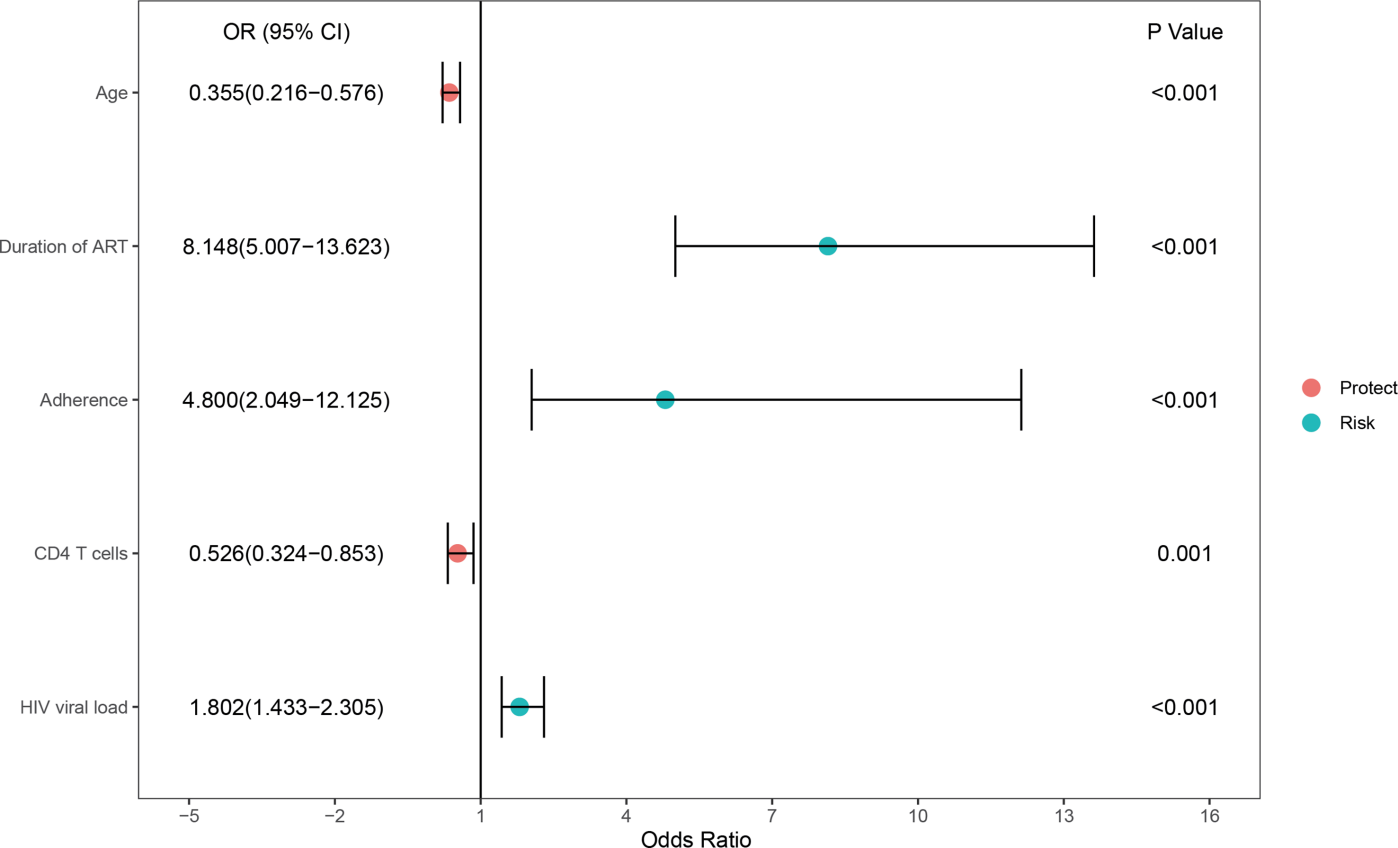
Forest plot of the significant parameters in the multivariable regression analysis.

In the training set, 33.5% (207/618) were HIV-DR patients, and most of the variables included in this study were significantly different between the DR and DS groups (**Supplementary Table**). To develop a highly accurate predictive model, LASSO regression was employed to select the most potent parameters (**Figure 2**). Finally, five independent risks, i.e., age, duration of ART, treatment adherence, CD4 T cell count and HIV viral load, were selected to establish the predictive model (**Figure 3**).

**Figure 4:**
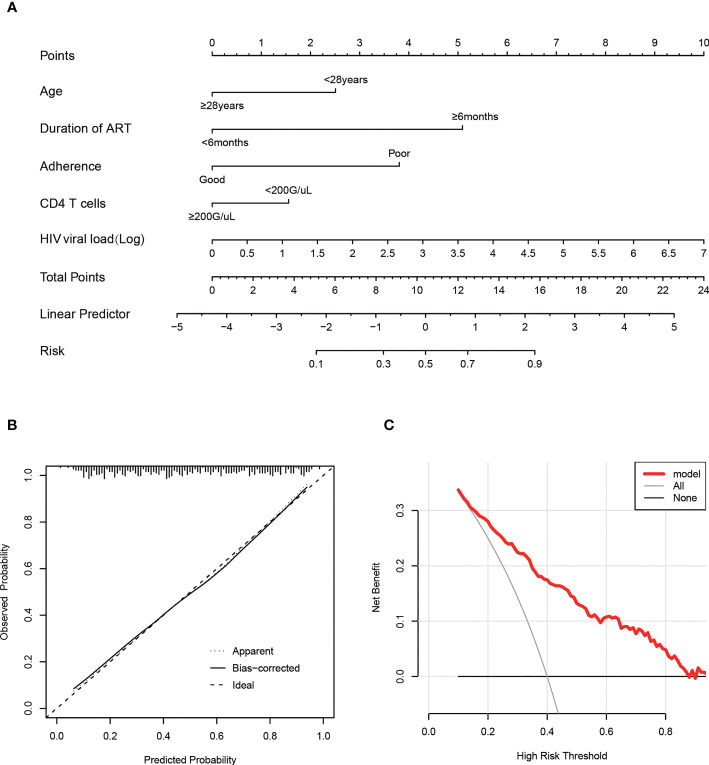
Calibration and clinical use of a predictive nomogram for the prediction of HIVDR in the train set. **(A)** Diagnostic nomogram for identifying HIVDR. **(B)** Calibration curve of the predictive nomogram. **(C)** DCA of the predictive nomogram.

And then, a nomogram (**Figure 4A**) based on the multivariable logistic model was generated, which showed a good calibration (**Figure 4B**). Decision curve analysis (DCA) was used to evaluate the clinical value of the diagnostic nomogram. As shown in **Figure 4C**, HIV patients would benefit more from utilizing this diagnostic nomogram than from acting on the all-or-none principle at a threshold probability of 0.3.

Furthermore, we transformed the nomogram into a scoring system with integer points to make this predictive model more accessible for doctors to utilize in clinical practice: age (2 points), duration of ART (5 points), adherence (4 points), CD4 T cells (1 point) and HIV viral load (1 point) (**Table 2**).

**Table 2:** A scoring system developed from a nomogram in the train set.

Variables	β(Absolute values)	Score generated from nomogram (points)	Score modified from nomogram (points)
Age(≥28y)	1.087	2.48	2
Time of therapy (≥6months)	2.289	5.22	5
Adherence (Poor)	1.633	3.73	4
CD4 T cells(≥200G/uL)	0.642	1.47	1
HIV viral load(log10)	0.626	1.43	1

### 3.3 Predictive effectiveness of the scoring system in the training set and validation set

**Figure 5:**
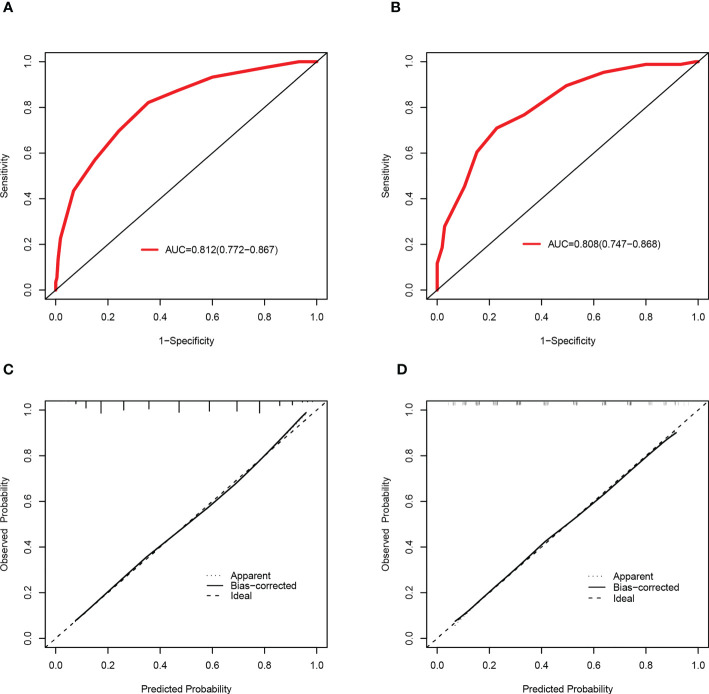
Discrimination and calibration of the scoring system for the prediction of HIVDR in the train and validation sets. ROC curves of the scoring system in the training set **(A)** and validation set **(B)**. Calibration curves of the scoring system in the training set **(C)** and validation set **(D)**.

Based on a cutoff value of 7.5 points, PLWH were more likely to be diagnosed with DR in the training set with a total number of points greater than 7.5, whereas they were less likely with a total number of points below 7.5. The corresponding sensitivity, specificity, PLR and NLR values for 7.5 points as the ideal cutoff were 82.13%, 64.55%, 2.32, and 0.28, respectively (**Table 3**). In addition, the AUC of this scoring system were 0.812(95%CI=0.772-0.867) and 0.808(95%CI=0.747-0.868) in the training set and validation set (**Figures 5A, B**), respectively. The scoring system also showed satisfactory calibration in both datasets. (**Figures 5C, D**).

**Table 3:** ROC analysis of the scoring system for identifying HIVDR.

Cut-off score	Youden index	Sensitivity	Specificity	PLR	NLR
>7.5	0.466	82.13(76.07-86.96)	64.55(57.79-70.78)	2.32(1.92-2.80)	0.28(0.21-0.37)
>8.5	0.455	87.44(81.96-91.48)	53.18(46.37-59.89)	1.87(1.61-2.17)	0.27(0.16-0.34)
>9.5	0.420	69.57(62.74-75.65)	75.91(69.60-81.29)	2.89(2.25-3.71)	0.40(0.33-0.49)
>6.5	0.406	57.00(49.95-63.79)	85.00(79.43-89.31)	3.80(2.72-5.32)	0.51(0.43-0.59)

HIVDR, HIV drug resistance; PLR, positive likelihood ratios; NLR, negative likelihood ratios.

## 4 Discussion

Although ART has achieved great progress in recent history (Palella et al., 1998), the increasing amount of drug resistant pathogens has caused therapeutic failure under low adherence and long duration of treatment (Zhang et al., 2020). In our study, the rate of drug resistance is 33.5%, with NNRTIs accounting for 45.2%, which is in concordance with other research (Beyrer and Pozniak, 2017). As transmitted or acquired DRMs constitute significant risk factors for ART effectiveness and AIDS therapy (Günthard et al., 2019; Li et al., 2021), HIV genotypic resistance testing has been advised for ART initiation, failure, and modification (Department of Health and Human Services, 2020). The development of DRM is a serious danger to the ongoing management of HIV replication as well as the possibly associated rise in viral strain transmission, which could increase the incidence of TDR (Tanaka et al., 2019; Santos-Pereira et al., 2021).

A first-line regimen for adults and adolescents was proposed in 2016 by World Health Organization, containing two nucleoside reverse-transcriptase inhibitors (NRTIs) with either a non-nucleoside reverse-transcriptase inhibitor (NNRTI) or an integrase inhibitor (INSTI) (World Health Organization, 2016). In 2021, WHO recommended DTG combination with an NRTI backbone as the preferred first-line regimen for PLHIV-infected individuals initiating ART (World Health Organization, 2021a).Many studies (Rocheleau et al., 2018; Crowell et al., 2021; Hackett et al., 2021; World Health Organization, 2021b) have shown that both NNRTIs and NRTIs induce high levels of HIV drug resistance among individuals with treatment failure (VL>1000 copies/ml), which collectively corroborate our results. In the present cohort, the DRM rates of NRTIs, NNRTIs, PIs and INSTIs were 24.8%, 45.2%, 0.2%, and 0%, respectively (**Figure 5**). These data also indicated that INSTIs represented by DTG induce a lower prevalence of drug resistance compared with NNRTIs. Furthermore, a study (McClung et al., 2022) determined a prevalence for transmitted drug-resistance mutations (TDRM) of 18.9% among individuals developing drug resistance within 3 months of treatment in the United States from 2014-2018. Based on the same criterion, we determined the proportion of DRM developed prior to treatment was 29.7%, and the main drugs causing resistance were NNRTIs and NRTIs.

Furthermore, most of the methods used to predict HIVDR are not sufficiently generalized for normal clinical practice due to inconvenience. Therefore, it is crucial to design a feasible and simple method to diagnose drug resistance. Based on several major parameters, we constructed a predictive model by selecting the most important indicators by the LASSO regression. This nomogram for predicting HIVDR in PLWH patients incorporated 5 variables, including age, duration of ART, adherence, CD4 T cells and HIV viral load, was built. In the training and validation sets, this nomogram had good calibration, diagnostic performance, and clinical utility. We transformed the nomogram into a scoring system for clinical application; this scoring system also demonstrated excellent diagnostic performance in the training and validation sets. It is important to note that our scoring method was based on a variety of clinical and laboratory indicators that are readily available in most hospitals, even community hospitals, with an acceptable overall cost.

In addition, we analyzed the clinical significance of these predictors. Regarding “duration of ART”, it is widely admitted that the longer the ART administration, the higher the risk of DRM. Nicholas (Nii-Trebi et al., 2013) found the duration of ART affects the development of DRM, and prolonged ART treatment increases the rates of virological failure (VF) and drug-resistant mutations. As shown in the current study, with a duration of treatment below 6 months, the incidence of DRM was 29.7% (90/303). Once the time surpassed 6 months, this incidence increased to 64.4% (203/315). Poor adherence (e.g., an irregular and unregulated use of medication) could have a significant impact on DRM. Evidence suggests PLWH face great challenges associated with poor ART adherence and HIV-1 drug resistance (Benson et al., 2020; Myer et al., 2020). Moreover, CD4 T cell count, serving as a crucial immunological indicator, could reflect immunological function and immune reconstitution partly; some studies also reported a negative association between elevated CD4 T cell count and HIV-MDR (Xiao et al., 2017; Lombardi et al., 2021). However, another study confirmed the opposite outcomes (Schultze et al., 2019), which might suggest CD4 count is affected by influencing factors, including incomplete immune reconstitution and acute inflammatory response. In addition, HIV viral load also contributed to the mutation of drug resistance genes. Jonah et al. (Omooja et al., 2019) found ADR prevalence could be as high as 73.2% among VF cases. In addition, studies also confirmed HIVDR in more than half of patients with VF (Tchouwa et al., 2018; Yan et al., 2022).

Interestingly, there were strong correlations (P<0.01) between drug resistance and some factors such as delayed treatment, Hb and BMI (**Table 1** and **Supplementary Table 1**), although the latter were not included in the prediction model by multivariable regression analysis. Their effects on DRM need further excavation and validation. Regarding treatment delay, we speculated that the earlier the ART initiation, the higher the CD4 T-cell count, the lower the HIV viral load and the better immune reconstitution, which may lead to a stable intra-organismal environment under antiviral therapy.

Our study has several advantages. Firstly, individual indicators either have low sensitivity or low specificity, and the combined indicators have better comprehensive predictive ability. Secondly, the indicators selected for the model are simple, convenient, rapid and inexpensive, which can be promoted and used in primary hospitals. Thirdly, a scoring model for facilitates clinical application is established to help clinicians identify HIV drug resistance high-risk groups and thus to determine them for gene testing, the gold standard of drug resistance. This way could reduce treatment failure and avoids wasting medical resources.

However, the patient selection in our study was biased and not randomized. To gain high-level evidence for the potential clinical applicability of the scoring system in the future, multicenter validation of the scoring system with a sizable research population is urgently required.

In conclusion, the novel scoring system is based on five easily accessible clinical parameters and shows excellent diagnostic performance and favorable calibration in determining the susceptibility to DRM in PLHIV. We recommend the widespread application of this novel scoring model in HIV-designated hospitals to identify patients at increased risk of DRM quickly and cost-effectively.

## Data availability statement

The original contributions presented in the study are included in the article/**Supplementary Material**. Further inquiries can be directed to the corresponding authors.

## Author contributions

LR and XW conceived and designed the experiments. JY and WZ wrote the article. JY, WZ, HL collected and analyzed the data. All authors contributed to the article and approved the submitted version.
