# Supplementary material for: Development and validation of a scoring system for the prediction of HIV drug resistance in Hubei province, China
Source: Front Cell Infect Microbiol. 2023 May 10;13:1147477. doi: 10.3389/fcimb.2023.1147477 (PMC10208424; doi:10.3389/fcimb.2023.1147477)
Supplement: Supplementary file 1 [file Table_1.docx]

**Supplementary table 1. Comparison of the clinical and laboratory features in training set**

| Parameters |  | DR(n=207) | DS(220) | p-value |
| --- | --- | --- | --- | --- |
| Age | <28years  ≥28years | 86(41.5%)  121(58.5%) | 59(26.8%)  161(73.2%) | 0.001** |
| Duration of therapy | <3months  ≥3months | 58(28%)  149(72%) | 145(65.9%)  75(34.1%) | <0.001*** |
| Delay of treatment | <1.4month  ≥1.4month | 127(61.4%)  80(38.6%) | 166(75.5%)  54(24.5%) | 0.002** |
| Treatment adherence | Good  Poor | 182(87.9%)  25(12.1%) | 211(95.3%)  9(4.1%) | 0.002** |
| SMZ-TMP Prophylaxis | No  Yes | 160(77.3)  47(22.7%) | 159(72.3%)  61(27.7%) | <0.233 |
| Medication regimen | Free  Other | 94(45.4%)  113(54.6%) | 103(46.8%)  117(53.2%) | 0.771 |
| CD4+ T cell count | <200 G/mL  ≥200 G/mL | 94(45.4%)  113(54.6%) | 58(26.4%)  162(73.6%) | <0.001*** |
| HIV-1 RNA levels | Mean log | 4.33(3.69-4.96) | 4.37(3.55-4.91) | <0.001*** |
| HIV-related symptoms and comorbidities | | | | |
|  | Fever>1m | 3(1.4%) | 14(6.4%) | 0.009** |
|  | Diarrhea>1m | 4(1.9%) | 11(5%) | 0.085 |
|  | Skin damage | 9(4.3) | 25(11.4) | 0.007** |
|  | PJP | 13(6.3%) | 3(1.4%) | 0.008** |
|  | TB or NTM | 2(1.0%) | 4(1.9%) | 0.686 |
|  | Others | 3(1.4%) | 16(7.3%) | 0.004** |
| Other laboratory parameters | | | | |
|  | WBC(10^9/L) | 5.19(3.91-6.41) | 5.32(4.33-6.64) | 0.123 |
|  | Hb(g/L) | 140(124-151) | 148(134-155) | <0.001*** |
|  | PLT(10^9/L) | 197(154-242) | 207(166-243) | 0.244 |
|  | Scr(μmol/L) | 66.4(59.2-78.3) | 68.2(60.4-76.2) | 0.428 |
|  | TG(mmol/L) | 1.48(0.94-2.56) | 1.51(1.01-2.44) | 0.941 |
|  | TC(mmol/L) | 4.16(3.47-4.88) | 4.15(3.61-4.85) | 0.606 |
|  | Bloodsugar(mmol/L) | 5.6(4.9-6.4) | 5.3(4.7-6.1) | 0.027* |
|  | ALT(U/L) | 23(15-38) | 26(18-40) | 0.086 |
|  | AST(U/L) | 27(21-31) | 27(21-33) | 0.589 |
|  | TBil(mol/L) | 12.2(9.3-16.0) | 13.2(9.6-16.2) | 0.528 |

**Notes**: **SMZ-TMP**, Compound Sulfamethoxazole; **Free medication regimen**: Lamivudine+ Efavirenz+Tenofovir; **TB**, tuberculosis; **NTM**, non-tuberculous mycobacteria; **PJP**, pneumocystis jirovecii pneumonia; **Other symptoms and comorbidities**: thrush，bacterial pneumonia, severe bacterial infection (except pneumonia), herpes simplex virus infection, herpes zoster, cytomegalovirus infection; **WBC**: white blood cell; **Hb**: haemoglobin; **PLT**: platelet; **Scr**: serum creatinine; **TG**: triglyceride; **TC**: total cholesterol; **ALT**: alanine aminotransferase; **AST**: artate aminotransferase; **TBil**: total bilirubin; *P<0.5, **P<0.01, ***P<0.001.
